# Supplementary material for: Self-assessment of unilateral and bimodal cochlear implant experiences in daily life
Source: PLoS One. 2020 Dec 3;15(12):e0242871. doi: 10.1371/journal.pone.0242871 (PMC7714204; doi:10.1371/journal.pone.0242871)
Supplement: S1 File — Qualitative questionnaire on bimodal hearing aid use and bimodal experiences for subjects who discarded the hearing aid (HA) aside the cochlear implant (CI) (UNI = unilateral group, n = 22) and subjects who continued to use the HA in the non-implanted ear (BIM = bimodal group, n = 26). Instructions, questions and results are presented. The origin of each question is described by its reference in literature. Results are presented per group as absolute frequencies as well as percentages across answering alternatives. The overall number of responding subjects per question may differ depending on the completeness of data as well as the relevance of each question in a patient-related manner. (PDF) [file pone.0242871.s002.pdf]

# BIMODAL QUESTIONNAIRE

BIM=bimodal users (continued to use the hearing aid aside the CI)
UNI=unilateral users (discarded the hearing aid aside the CI)

**Instructions:** By this questionnaire we hope to gain more insight into the advantages and disadvantages of wearing a hearing aid and the reasons why patients do (not) continue to wear a hearing aid. Please answer each question by checking the box that best represents your situation, experience or vision. Feel free to add any comments in the assigned free space or on the back of the page (please add the number of the question).

Remember the period **BEFORE** you received a cochlear implant. These questions ask about your experiences with hearing aids during this period.

Question based on....

*1. When did you start wearing hearing aids? (Please indicate year and month if possible)*

Right: ☐ Left: ☐

*2. When did you stop wearing hearing aids? (Please indicate year and month or "I continued to use a hearing aid untill surgery")*

Right: ☐ Left: ☐

*3. How much did you wear your hearing aid(s) before your surgery?* — *Fitzpatrick et al. (2010)*

| | | | Frequency | | Percentage | | % Bars | |
|---|---|---|---|---|---|---|---|---|
| | | | BIM | UNI | BIM | UNI | BIM | UNI |
| Both sides: | more than 10 hours a day | (almost ) always | 17 | 13 | 68,0 | 61,9 | | |
| | 5 to 10 hours a day | sometimes | 1 | 2 | 4,0 | 9,5 | | |
| | less than 5 hours a day | rarely | 0 | 1 | 0,0 | 4,8 | | |
| | | never | 7 | 5 | 28,0 | 23,8 | | |
| Right: | more than 10 hours a day | (almost ) always | 2 | 2 | 8,0 | 9,5 | | |
| | 5 to 10 hours a day | sometimes | 0 | 0 | 0,0 | 0,0 | | |
| | less than 5 hours a day | rarely | 0 | 0 | 0,0 | 0,0 | | |
| | | never | 23 | 19 | 92,0 | 90,5 | | |
| Left: | more than 10 hours a day | (almost ) always | 5 | 2 | 20,0 | 9,5 | | |
| | 5 to 10 hours a day | sometimes | 0 | 1 | 0,0 | 4,8 | | |
| | less than 5 hours a day | rarely | 0 | 0 | 0,0 | 0,0 | | |
| | | never | 20 | 18 | 80,0 | 85,7 | | |

*4. Could you hear environmental sounds with your hearing aid(s) before your surgery?* — *Fitzpatrick et al. (2010)*

| | Frequency | | Percentage | | % Bars | |
|---|---|---|---|---|---|---|
| | BIM | UNI | BIM | UNI | BIM | UNI |
| yes | 18 | 19 | 72,0 | 86,4 | | |
| no | 7 | 3 | 28,0 | 13,6 | | |

*5. Could you understand people talking to you with your hearing aid(s) before your surgery?* — *Fitzpatrick et al. (2010)*

| | | Frequency | | Percentage | | % Bars | |
|---|---|---|---|---|---|---|---|
| | | BIM | UNI | BIM | UNI | BIM | UNI |
| With lipreading: | yes | 22 | 18 | 88,0 | 81,8 | | |
| | no | 3 | 4 | 12,0 | 18,2 | | |
| Without lipreading: | yes | 7 | 5 | 28,0 | 22,7 | | |
| | no | 18 | 17 | 72,0 | 77,3 | | |

*6. All in all, how helpful was your hearing aid(s)?* — *Fitzpatrick et al. (2010)*

| | Frequency | | Percentage | | % Bars | |
|---|---|---|---|---|---|---|
| | BIM | UNI | BIM | UNI | BIM | UNI |
| very helpful | 14 | 6 | 56,0 | 27,3 | | |
| somewhat helpful | 10 | 6 | 40,0 | 27,3 | | |
| rarely helpful | 1 | 8 | 4,0 | 36,4 | | |
| not at all helpful | 0 | 2 | 0,0 | 9,1 | | |

*7. How do you feel that you coped with your hearing loss before you received your imlant?* — *Fitzpatrick et al. (2010)*

| | Frequency | | Percentage | | % Bars | |
|---|---|---|---|---|---|---|
| | BIM | UNI | BIM | UNI | BIM | UNI |
| well all of the time | 2 | 3 | 8,0 | 13,6 | | |
| well much of the time | 11 | 9 | 44,0 | 40,9 | | |
| well some of the time | 7 | 7 | 28,0 | 31,8 | | |
| not well at all (it really affected me negatively) | 5 | 3 | 20,0 | 13,6 | | |

The following questions deal with your decision process about using a hearing aid with the cochlear implant.

*8. Had you made a decision before your surgery about whether you would use your hearing aid with your cochlear implant?* — *Fitzpatrick et al. (2010)*

| | Frequency | | Percentage | | % Bars | |
|---|---|---|---|---|---|---|
| | BIM | UNI | BIM | UNI | BIM | UNI |
| yes | 17 | 7 | 65,4 | 31,8 | | |
| no | 9 | 15 | 34,6 | 68,2 | | |

*9. Did your audiologist talk to you about the possibility of wearing your hearing aid with your cochlear implant?* *Fitzpatrick et al. (2010)*

| | Frequency BIM | Frequency UNI | Percentage BIM | Percentage UNI | % Bars BIM | % Bars UNI |
|---|---|---|---|---|---|---|
| yes, before surgery | 9 | 7 | 34,6 | 31,8 | | |
| yes, after surgery | 2 | 4 | 7,7 | 18,2 | | |
| yes, both before and after surgery | 6 | 3 | 23,1 | 13,6 | | |
| no, use of a hearing aid was not discussed | 3 | 6 | 11,5 | 27,3 | | |
| I can't remember | 6 | 2 | 23,1 | 9,1 | | |

*10. Did your audiologist encourage you to try wearing your hearing aid with your cochlear implant?* *Fitzpatrick et al. (2010)*

| | Frequency BIM | Frequency UNI | Percentage BIM | Percentage UNI | % Bars BIM | % Bars UNI |
|---|---|---|---|---|---|---|
| yes, before surgery | 6 | 1 | 23,1 | 4,5 | | |
| yes, after surgery | 5 | 4 | 19,2 | 18,2 | | |
| yes, both before and after surgery | 4 | 4 | 15,4 | 18,2 | | |
| no | 7 | 9 | 26,9 | 40,9 | | |
| I can't remember | 4 | 4 | 15,4 | 18,2 | | |

The following questions deal with your experiences of using a hearing aid **AFTER** you received the cochlear implant.

*11. Has the hearing in your un-implanted ear changed since you received your cochlear implant?* *Fitzpatrick et al. (2009)*

| | Frequency BIM | Frequency UNI | Percentage BIM | Percentage UNI | % Bars BIM | % Bars UNI |
|---|---|---|---|---|---|---|
| don't notice any difference | 8 | 11 | 30,8 | 50,0 | | |
| seems a little worse | 10 | 2 | 38,5 | 9,1 | | |
| seems a little better | 4 | 1 | 15,4 | 4,5 | | |
| seems a lot worse | 3 | 6 | 11,5 | 27,3 | | |
| seems a lot better | 1 | 2 | 3,8 | 9,1 | | |

*12. How often do you use a hearing aid with your cochlear implant?* *Fitzpatrick et al. (2009)*

| | | Frequency BIM | Frequency UNI | Percentage BIM | Percentage UNI | % Bars BIM | % Bars UNI |
|---|---|---|---|---|---|---|---|
| more than 10 hours a day | (almost ) always | 21 | 0 | 80,8 | 0,0 | | |
| 5 to 10 hours a day | sometimes | 2 | 0 | 7,7 | 0,0 | | |
| less than 5 hours a day | rarely | 3 | 0 | 11,5 | 0,0 | | |
| | never | 0 | 22 | 0,0 | 100,0 | | |

*13. How often do you use only your cochlear implant?* *Fitzpatrick et al. (2009)*

| | | Frequency BIM | Frequency UNI | Percentage BIM | Percentage UNI | % Bars BIM | % Bars UNI |
|---|---|---|---|---|---|---|---|
| more than 10 hours a day | (almost ) always | 2 | 22 | 8,0 | 100,0 | | |
| 5 to 10 hours a day | sometimes | 2 | 0 | 8,0 | 0,0 | | |
| less than 5 hours a day | rarely | 7 | 0 | 28,0 | 0,0 | | |
| | never | 14 | 0 | 56,0 | 0,0 | | |

*14. How often do you use only a hearing aid?* *Fitzpatrick et al. (2009)*

| | | Frequency BIM | Frequency UNI | Percentage BIM | Percentage UNI | % Bars BIM | % Bars UNI |
|---|---|---|---|---|---|---|---|
| more than 10 hours a day | (almost ) always | 2 | 0 | 8,0 | 0,0 | | |
| 5 to 10 hours a day | sometimes | 1 | 0 | 4,0 | 0,0 | | |
| less than 5 hours a day | rarely | 6 | 0 | 24,0 | 0,0 | | |
| | never | 16 | 22 | 64,0 | 100,0 | | |

*15. When did you start using your hearing aid and cochlear implant together?* *Fitzpatrick et al. (2009)*

| | Frequency BIM | Frequency UNI | Percentage BIM | Percentage UNI | % Bars BIM | % Bars UNI |
|---|---|---|---|---|---|---|
| right away | 20 | 6 | 76,9 | 27,3 | | |
| within 1 to 3 months of the implant | 2 | 2 | 7,7 | 9,1 | | |
| within 3 to 6 months of the implant | 2 | 1 | 7,7 | 4,5 | | |
| after the first 6 months of the implant | 2 | 2 | 7,7 | 9,1 | | |
| I never tried a hearing aid with the implant | 0 | 11 | 0,0 | 50,0 | | |

*--> If you never tried a hearing aid with your cochlear implant: go straight on to question 38.*

*16. After receiving your implant, how long did you use your hearing aid in the other ear?* *Fitzpatrick et al. (2010)*

| | Frequency BIM | Frequency UNI | Percentage BIM | Percentage UNI | % Bars BIM | % Bars UNI |
|---|---|---|---|---|---|---|
| I still use my hearing aid in the other ear | 26 | 0 | 100,0 | 0,0 | | |
| I do no longer use a hearing aid in the other ear, but I did try a hearing aid for: | | | | | | |
| more than 6 months | 0 | 4 | 0,0 | 36,4 | | |
| 1 to 6 months | 0 | 2 | 0,0 | 18,2 | | |
| 1 to 4 weeks | 0 | 1 | 0,0 | 9,1 | | |
| 1 to 7 days | 0 | 2 | 0,0 | 18,2 | | |
| less than 1 day | 0 | 2 | 0,0 | 18,2 | | |

*--> Please answer the following questions, even if you no longer use a hearing aid aside your implant. You will be asked about your experiences during the period that you did try the hearing aid.*

*17. How fast did you adapt to using your hearing aid and cochlear implant together?* *Fitzpatrick et al. (2009)*

| | Frequency | | Percentage | | % Bars | |
|---|---|---|---|---|---|---|
| | BIM | UNI | BIM | UNI | BIM | UNI |
| right away | 18 | 4 | 69,2 | 40,0 | | |
| within 1 to 3 months of the implant | 4 | 1 | 15,4 | 10,0 | | |
| within 3 to 6 months of the implant | 2 | 0 | 7,7 | 0,0 | | |
| after the first 6 months of the imlant | 1 | 0 | 3,8 | 0,0 | | |
| I couldn't get used to the hearing aid | 1 | 5 | 3,8 | 50,0 | | |

*18. The hearing aid that you used with your cochlear implant, is this the same hearing aid you used before your surgery?* *Fitzpatrick et al. (2009)* *Fitzpatrick et al. (2010)*

| | Frequency | | Percentage | | % Bars | |
|---|---|---|---|---|---|---|
| | BIM | UNI | BIM | UNI | BIM | UNI |
| yes | 13 | 9 | 50,0 | 90,0 | | |
| no, I immediately started with a new hearing aid after surgery | 3 | 1 | 11,5 | 10,0 | | |
| no, first I used the old hearing aid, but now I have a new hearing aid since...... | 10 | 0 | 38,5 | 0,0 | | |

*19. How old was the hearing aid that you used with your cochlear implant?* *Fitzpatrick et al. (2010)*

| | Frequency | | Percentage | | % Bars | |
|---|---|---|---|---|---|---|
| | BIM | UNI | BIM | UNI | BIM | UNI |
| more than 10 years old | 0 | 1 | 0,0 | 11,1 | | |
| 5 to 10 years old | 6 | 2 | 23,1 | 22,2 | | |
| 3 to 5 years old | 10 | 4 | 38,5 | 44,4 | | |
| less than 3 years old | 10 | 2 | 38,5 | 22,2 | | |

*20. Did you need to have your hearing aid adjusted after using your cochlear implant?* *Fitzpatrick et al. (2009)* *Fitzpatrick et al. (2010)*

| | Frequency | | Percentage | | % Bars | |
|---|---|---|---|---|---|---|
| | BIM | UNI | BIM | UNI | BIM | UNI |
| not at all, the same settings that were in place before my surgery were used | 17 | 6 | 65,4 | 66,7 | | |
| yes, the fitting was changed: 1 to 3 times | 5 | 1 | 19,2 | 11,1 | | |
| 3 to 6 times | 1 | 2 | 3,8 | 22,2 | | |
| more than 6 times | 3 | 0 | 11,5 | 0,0 | | |
| These changes were made by: | | | | | | |
| the audiologist (audiological centre) | 4 | 0 | 44,4 | 0,0 | | |
| the hearing aid dispenser (shop) | 5 | 2 | 55,6 | 100,0 | | |

*21. How did you set the volume of your hearing aid and cochlear implant when whorn combined? Please check all that apply.* *Fitzpatrick et al. (2009)*

| | Frequency | |
|---|---|---|
| Hearing aid: | BIM | UNI |
| the same as when I use the hearing aid alone | 17 | 3 |
| different compared to when I use the hearing aid alone | 2 | 2 |
| depends on the situation | 6 | 2 |
| I rarely use the volume control on my hearing aid | 8 | 3 |
| I didn't have access to the volume control of my hearing aid | 1 | 1 |
| Cochlear implant: | | |
| the same as when I use the cochlear implant alone | 17 | 5 |
| different compared to when I use the cochlear implant alone | 0 | 1 |
| depends on the situation | 7 | 2 |
| I rarely use the volume control on my cochlear implant | 7 | 1 |
| I didn't have access to the volume control of my cochlear implant | 1 | 1 |

Percentages cannot be calculated since more than one alternative was possible.

Percentages cannot be calculated since more than one alternative was possible.

*22. How did you adjust the program of your hearing aid and the cochlear implant when whorn together? Please check all that apply.* *Fitzpatrick et al. (2009)*

| | Frequency | |
|---|---|---|
| Hearing aid: | BIM | UNI |
| the same as when I use the hearing aid alone | 18 | 5 |
| different compared to when I use the hearing aid alone | 0 | 0 |
| depends on the situation | 5 | 2 |
| I rarely use different programs on my hearing aid | 5 | 2 |
| I don't have access to the program function of my hearing aid | 2 | 1 |
| Cochlear implant: | | |
| the same as when I use the cochlear implant alone | 15 | 7 |
| different compared to when I use the cochlear implant alone | 1 | 1 |
| depends on the situation | 10 | 3 |
| I rarely use different programs on my cochlear implant | 6 | 0 |
| I don't have access to the program function of my cochlear implant | 1 | 0 |

Percentages cannot be calculated since more than one alternative was possible.

Percentages cannot be calculated since more than one alternative was possible.

*23. In which of the following settings did you wear your hearing aid with your cochlear implant? Please check all that apply.* *Fitzpatrick et al. (2010)*

| | Frequency | |
|---|---|---|
| | BIM | UNI |
| I use(d) my hearing aid at work | 1 | 0 |
| I use(d) my hearing aid at home | 3 | 5 |
| I use(d) my hearing aid everywhere I went | 23 | 5 |

Percentages cannot be calculated since more than one alternative was possible.

*24. In wich of the following listening conditions did you wear your hearing aid with your cochlear implant? Please check all that apply.* *Fitzpatrick et al. (2010)*

| | Frequency | |
|---|---|---|
| | BIM | UNI |
| I use(d) my hearing aid in noisy situations | 22 | 4 |
| I use(d) my hearing aid in quiet situations | 25 | 9 |
| I use(d) my hearing aid when listening to music | 22 | 5 |
| other listening situations: .... | 0 | 0 |

Percentages cannot be calculated since more than one alternative was possible.

*25. In quiet listening situations (e.g. a 1-on-1 conversation), you hear better with your:* *Fitzpatrick et al. (2009)*

| | Frequency | | Percentage | |
|---|---|---|---|---|
| | BIM | UNI | BIM | UNI |
| cochlear implant only | 0 | 6 | 0,0 | 60,0 |
| hearing aid only | 0 | 0 | 0,0 | 0,0 |
| cochlear implant and hearing aid together | 25 | 0 | 100,0 | 0,0 |
| no difference with any of the above | 0 | 4 | 0,0 | 40,0 |

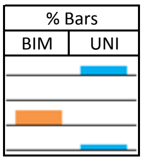


*26. In noisy situations (e.g. in a restaurant, at a party, shopping center, in a large group discussion), you hear better with your:* *Fitzpatrick et al. (2009)*

| | Frequency | | Percentage | |
|---|---|---|---|---|
| | BIM | UNI | BIM | UNI |
| cochlear implant only | 0 | 5 | 0,0 | 55,6 |
| hearing aid only | 2 | 0 | 7,7 | 0,0 |
| cochlear implant and hearing aid together | 20 | 2 | 76,9 | 22,2 |
| no difference with any of the above | 4 | 2 | 15,4 | 22,2 |

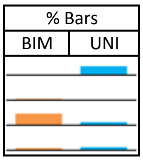


*27. In places with an echo (e.g. church, auditorium), you hear better with your:* *Fitzpatrick et al. (2009)*

| | Frequency | | Percentage | |
|---|---|---|---|---|
| | BIM | UNI | BIM | UNI |
| cochlear implant only | 2 | 6 | 7,7 | 85,7 |
| hearing aid only | 1 | 0 | 3,8 | 0,0 |
| cochlear implant and hearing aid together | 15 | 0 | 57,7 | 0,0 |
| no difference with any of the above | 8 | 1 | 30,8 | 14,3 |

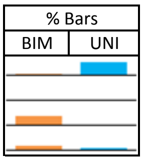


*28. Music sounds best with your:* *Fitzpatrick et al. (2009)*

| | Frequency | | Percentage | |
|---|---|---|---|---|
| | BIM | UNI | BIM | UNI |
| cochlear implant only | 1 | 6 | 3,8 | 85,7 |
| hearing aid only | 1 | 0 | 3,8 | 0,0 |
| cochlear implant and hearing aid together | 20 | 0 | 76,9 | 0,0 |
| no difference with any of the above | 4 | 1 | 15,4 | 14,3 |

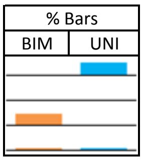


*29. Telling from which direction souds are coming is easier with your:* *Fitzpatrick et al. (2009)*

| | Frequency | | Percentage | |
|---|---|---|---|---|
| | BIM | UNI | BIM | UNI |
| cochlear implant only | 2 | 4 | 7,7 | 50,0 |
| hearing aid only | 1 | 0 | 3,8 | 0,0 |
| cochlear implant and hearing aid together | 13 | 1 | 50,0 | 12,5 |
| no difference with any of the above | 10 | 3 | 38,5 | 37,5 |

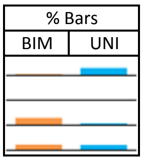


*30. Are there certain sounds that you hear better (or worse) when wearing your hearing aid and cochlear implant together? Describe which sounds, e.g. sounds in the environment (e.g. telephone, water), speech sounds (e.g. consonants, vowels, quiet voices, etc.)* *Fitzpatrick et al. (2009)*

*31. Please use the space below and the reverse side of this page to share any additional information about how you feel that using a hearing aid and cochlear implant together helps or hinders listening and communication.* *Fitzpatrick et al. (2009)*

*32. When you are using your hearing aid and cochlear implant together, where do you perceive sound?* *Tyler et al. (2002)*

| | Frequency | | Percentage | |
|---|---|---|---|---|
| | BIM | UNI | BIM | UNI |
| in the middle of your head | 8 | 0 | 34,8 | 0,0 |
| closer to the the ear with cochlear implant | 8 | 3 | 34,8 | 33,3 |
| closer to the ear with hearing aid | 1 | 0 | 4,3 | 0,0 |
| separatly in both ears | 5 | 5 | 21,7 | 55,6 |
| depends on the situation | 1 | 1 | 4,3 | 11,1 |

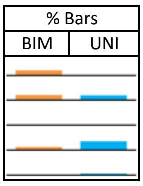


*33. When you are using your hearing aid and cochlear implant together, where is the sound louder?* *Tyler et al. (2002)*

| | Frequency | | Percentage | |
|---|---|---|---|---|
| | BIM | UNI | BIM | UNI |
| in the cochlear implant ear | 15 | 7 | 65,2 | 77,8 |
| in the hearing aid ear | 1 | 1 | 4,3 | 11,1 |
| same loudness in both ears | 7 | 1 | 30,4 | 11,1 |
| depends on the situation | 0 | 0 | 0,0 | 0,0 |

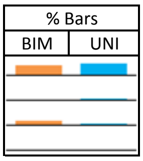


*34. When using the cochlear implant and hearing aid together, does the sound "fuse" or "join" to become one image?* *Tyler et al. (2002)*

| | Frequency | | Percentage | |
|---|---|---|---|---|
| | BIM | UNI | BIM | UNI |
| yes, it fuses together to become one image | 16 | 1 | 66,7 | 11,1 |
| no, I hear two separate images | 5 | 7 | 20,8 | 77,8 |
| different, namely: .... | 3 | 1 | 12,5 | 11,1 |

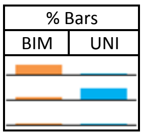

*35. Which ear contributes most to understanding speech?*

| | Frequency | | Percentage | | % Bars | |
|---|---|---|---|---|---|---|
| | BIM | UNI | BIM | UNI | BIM | UNI |
| especially the cochlear implant ear | 12 | 10 | 46,2 | 100,0 | | |
| rather the cochlear implant ear | 2 | 0 | 7,7 | 0,0 | | |
| the hearing aid and cochlear implant ear add equally | 9 | 0 | 34,6 | 0,0 | | |
| rather the hearing aid ear | 1 | 0 | 3,8 | 0,0 | | |
| especially the hearing aid ear | 2 | 0 | 7,7 | 0,0 | | |

*36. Considering everything, do you think your hearing aside the cochlear implant is worth the trouble?* *Cox et al. (2002)*

| | Frequency | | Percentage | | % Bars | |
|---|---|---|---|---|---|---|
| | BIM | UNI | BIM | UNI | BIM | UNI |
| no, not worthwhile at all | 1 | 7 | 3,8 | 77,8 | | |
| a bit worthwhile | 2 | 1 | 7,7 | 11,1 | | |
| reasonably worthwhile | 4 | 0 | 15,4 | 0,0 | | |
| really worthwhile | 8 | 1 | 30,8 | 11,1 | | |
| extremely worthwhile | 11 | 0 | 42,3 | 0,0 | | |

*37. Considering everything, how much has your hearing aid aside the cochlear implant changed your enjoyment in life?* *Cox et al. (2002)*

| | Frequency | | Percentage | | % Bars | |
|---|---|---|---|---|---|---|
| | BIM | UNI | BIM | UNI | BIM | UNI |
| worse | 0 | 3 | 0,0 | 33,3 | | |
| no difference | 2 | 5 | 7,7 | 55,6 | | |
| a bit better | 5 | 0 | 19,2 | 0,0 | | |
| better | 7 | 0 | 26,9 | 0,0 | | |
| much better | 12 | 1 | 46,2 | 11,1 | | |

*38. List the 3 factors that most influenced your decision (not) to use year hearing aid with the cochlear implant (from 1 to 3 in order of importance).* *Fitzpatrick et al. (2010)*

| | |
|---|---|
| 1. | |
| 2. | |
| 3. | |

*39. Please feel free to share any additional information concerning the reasons why you decided (not) to wear your hearing aid in conjunction with your* *Fitzpatrick et al. (2010)*


Bibliography

Cox, R. M., Stephens, D., & Kramer, S. E. (2002). Translations of the International Outcome inventory for Hearing Aids (IOI-HA)*International Journal of Audiology* , *41* (1), 3–26.

Fitzpatrick, E. M., & Leblanc, S. (2010). Exploring the factors influencing discontinued hearing aid use in patients with unilateral cochlear implants*Trends in Amplification* , *14* (4), 199–210.

Fitzpatrick, E. M., Seguin, C., Schramm, D., Chenier, J., & Armstrong, S. (2009). Users' experience of a cochlear implant combined with a hearing aid*Int J Audiol* , *48* (4), 172–182.

Tyler, R. S., Parkinson, A. J., Wilson, B. S., Witt, S., Preece, J. P., & Noble, W. (2002). Patients utilizing a hearing aid and a cochlear implant: speech perception and localization*Ear and Hearing* , *23* (2), 98–105.
