# Supplementary figures and images for: Self-assessment of unilateral and bimodal cochlear implant experiences in daily life
Source: PLoS One. 2020 Dec 3;15(12):e0242871. doi: 10.1371/journal.pone.0242871 (PMC7714204; doi:10.1371/journal.pone.0242871)

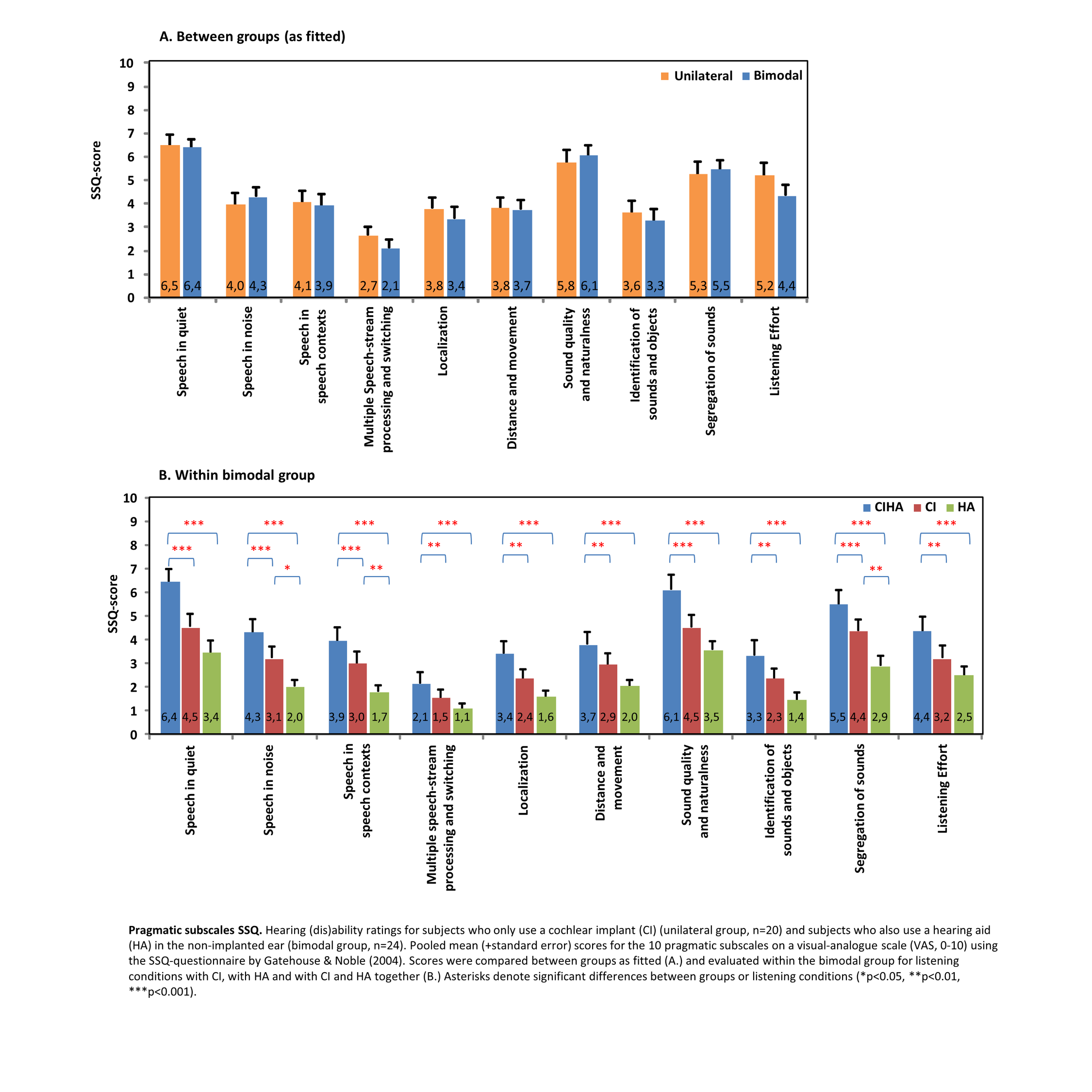

Supplement: S1 Fig — Hearing (dis)ability ratings for subjects who only use a cochlear implant (CI) (unilateral group, n = 20) and subjects who also use a hearing aid (HA) in the non-implanted ear (bimodal group, n = 24). Pooled mean (+standard error) scores for the 10 pragmatic subscales on a visual-analogue scale (VAS, 0–10) using the SSQ-questionnaire by Gatehouse & Noble [37]. Scores were compared between groups as fitted (A.) and evaluated within the bimodal group for listening conditions with CI, with HA and with CI and HA together (B.) Asterisks denote significant differences between groups or listening conditions (*p<0.05, **p<0.01, ***p<0.001). (TIF) [file pone.0242871.s003.tif]
